# Effect of different timings of umbilical cord clamping on the level of CD34^+^ cells in full-term neonates

**DOI:** 10.1038/s41598-023-50100-9

**Published:** 2023-12-21

**Authors:** Mai S. Korkor, Mohamed khashaba, Sara A. Mohamed, Ahmad Darwish

**Affiliations:** 1 Pediatric Department, Mansoura University Children’s Hospital, Faculty of Medicine, Mansoura University, Mansoura, Egypt; 2 Neonatology Unit, Pediatric Department, Faculty of Medicine, Mansoura University, Mansoura, Egypt; 3 Obstetric and Gynecology Department, Faculty of Medicine, Mansoura University, Mansoura, Egypt; 4 Mansoura Research Center for Cord Stem Cells (MARC-CSC), Faculty of Medicine, Mansoura University, Mansoura, Egypt; 5 Hematology/Oncology/Bone Marrow Transplantation Unit, Pediatric Department, Faculty of Medicine, Mansoura University, Mansoura, Egypt

**Keywords:** Stem cells, Health care, Medical research

## Abstract

Despite the fact that delayed cord clamping (DCC) is recommended by many international organizations, early cord clamping is still widely practiced worldwide. The overarching goal of the DCC practice is to maximize neonatal benefits as achieving higher hemoglobin levels and decreasing the incidence of anemia as well as avoiding the adverse consequences. The current study was conducted to identify the effect of of DCC on the number of CD34^+^ stem cells in cord blood of full term neonates after two different timings (30 and 60 s after birth). One hundred and three full-term (FT) newborn babies (gestational age 37–40 weeks) delivered by elective cesarean section were randomly assigned into 2 groups: Group 1: babies were subjected to DCC 30 s after birth (50 newborns). Group 2: babies were subjected to DCC 60 s after birth (53 newborns). Neonates in group 2 had significantly higher levels of hemoglobin, hematocrit, total nucleated cells and CD34^+^ cells compared to those in group 1. The practice of DCC 60 s after birth achieved better CD34^+^ stem cells transfer in FT neonates than clamping the cord after 30 s.

## Introduction

The optimal timing of umbilical cord clamping is controversial in both preterm (PT) and full term (FT) neonates^1^*.* Although it has been concluded from many randomized clinical trials (RCT) that delayed cord clamping (DCC) for 30–180 s after birth in FT infants and for at least 30 s in PT infants has significant health benefits, this practice is not widely used because of the concerns that such a delay might prevent timely resuscitation^2^. However, resuscitation with an intact cord allows the infant to be supported physiologically through placental respiration while resuscitation is underway^3^. All the neonatal resuscitation program (NRP) recommendations can be followed while the infant is still attached to the placenta^4,5^.

American College of Obstetricians and Gynecologists (ACOG) supports DCC in PT infants, when feasible (for 30–60 s). There is insufficient evidence to confirm the potential benefits of DCC in FT infants^6^. However, American Heart Association (AHA) recommends DCC for 30 s in both FT and PT infants who do not require resuscitation at birth^7^. Benefits of DCC in FT infants include higher hemoglobin (HB) and hematocrit (Hct) in the early neonatal period, higher total body iron stores and circulating ferritin level at 2–4 months of age and lower incidence of iron-deficiency anemia around 4 months of age^8^. In addition, fetal blood is highly enriched with CD34 + cells; marker of hematopoietic stem cells (HSC), and multipotent lineage stem cells which have a great potential for regeneration and repair of organ damage*.* Iatrogenic loss of stem cells at birth through early cord clamping (ECC) could predispose infants to diseases such as chronic lung disease, asthma, diabetes, cerebral palsy, infection, and neoplasm^9^.

In terms of adverse outcome of DCC, it includes higher peak values of bilirubin during the first week after birth especially in PT infant and increased need for phototherapy in both PT and FT infants, but this may be related to other associated pathologic issues especially with PT babies for example; hypoxia, hypoglycemia and poor feeding^10^.

One of the current problems facing the practice of DCC is the emerging need for cord blood to supply cord blood banks. DCC leads to marked decrease of the obtained blood volume and the available number of hematopoietic precursor cells, which may not be sufficient for the therapeutic applications^11^. However, this issue is still debatable^12,13^ and the timing of umbilical cord clamping should not be altered for the purpose of collecting cord blood for banking^14^ as new techniques as ex vivo expansions of stem cells or accelerating its homing to the bone marrow can overcome this problem^15–17^. We conducted the current study to investigate the effect of DCC (after 30 and 60 s) on CD34^+^ stem cells in FT babies.

## Subjects and methods

The present study was conducted at the delivery room and in the Obstetrics & Gynecology department in Mansoura University Hospital (MUH). All necessary Maternal data were obtained from medical records (Such as name, age, occupation, residency, gravidity, parity, medical history, pregnancy related complications, blood pressure, blood glucose monitoring during pregnancy, antenatal screening for various diseases and serologic results for blood transmitted diseases (syphilis, Hepatitis B virus (HBV), Hepatitis C virus (HCV), Human Immunodeficiency Virus (HIV), maternal blood group, ABO and Rh incompatibility, maternal hemoglobin and need for blood transfusion antenatal or intra-partum and use of antenatal steroids.

The indication of elective cesarean section (ECS) and Doppler evaluation of the placenta and the cord were revised. Potentially eligible mothers were approached upon arrival and written informed consent was obtained before enrollment in the study after a thorough explanation of the procedure.

### Study designs

This was a cross sectional study with analytic components.

### Participants

Random selection of 103 eligible newborn babies were enrolled in the study.

### Inclusion criteria

Full term babies (gestational age 37–40 weeks), delivered by elective cesarean section (ECS) and didn’t require more than supportive care (vigorous babies with normal heart rate and breathing) were eligible for the study.

### Exclusion criteria

Preterm newborns (gestational age less than 37 weeks), multiple gestation and babies who required major resuscitative measures along with presence of any maternal illnesses as diabetes or hypertension or blood transmitted diseases (syphilis, HBV, HCV, HIV) were excluded. Additionally, those with placental abnormalities and intrauterine growth restriction (IUGR) as evident by ultrasound fetal biometry and uterine Doppler studies, where delay of resuscitation measures was not possible, were also excluded from the study.

### Intervention

The intervention was to delay clamping of the umbilical cord (30 s or 60 s after delivery). All aspects of obstetric and neonatal care were managed according to the standard protocol of MUH, Mansoura, Egypt. All staff in the delivery unit were fully aware of the study procedure before the trial started.

Immediately after transferring the baby to the adjacent ordinary care unit, every baby went through all steps of newborn ordinary care, dried thoroughly under radiant warmer (servo control), maintained in sniffing position with brief gentle suction as needed. The time from complete delivery of the baby to the first clamp applied on the umbilical cord was measured with a stopwatch by the assistant. Consequently, the umbilical cord was sterilized with 70% alcohol swabs. Then, two ml of cord blood were withdrawn within 2–5 min after delivery by using wide bore needle in K2 EDTA tube (Greiner Bio One), then gently mixed by inverting the tube 5–10 times and placed on a rocker for up to 30 min, and eventually refrigerated at 2–8 °C. Clotted samples were discarded.

The collected samples were analyzed at Mansoura Research Center for Cord Stem Cells (MARC-CSC). Samples had to be analyzed within 12 h of blood collection to ensure viability of stem cells. Neonatal data such as gestational age, gender and birth weight were recorded in data sheets.

### Outcomes

The primary outcome was to measure the total nucleated cells (TNC) and peripheral blood percentage of cluster of differentiation 34 (CD34^+^) in cord blood sample as a marker of stem cell level. The TNC count which was determined using the automated cell counter, sysmex XS-800i cell counter (Sysmex Corporation, JAPAN) and the number of hematopoietic stem cells CD34^+^ which was evaluated by flow cytometric analysis by BD Accuri™ C6 Cytometer (Becton, Dickinson, and Company).

**Figure 1:**
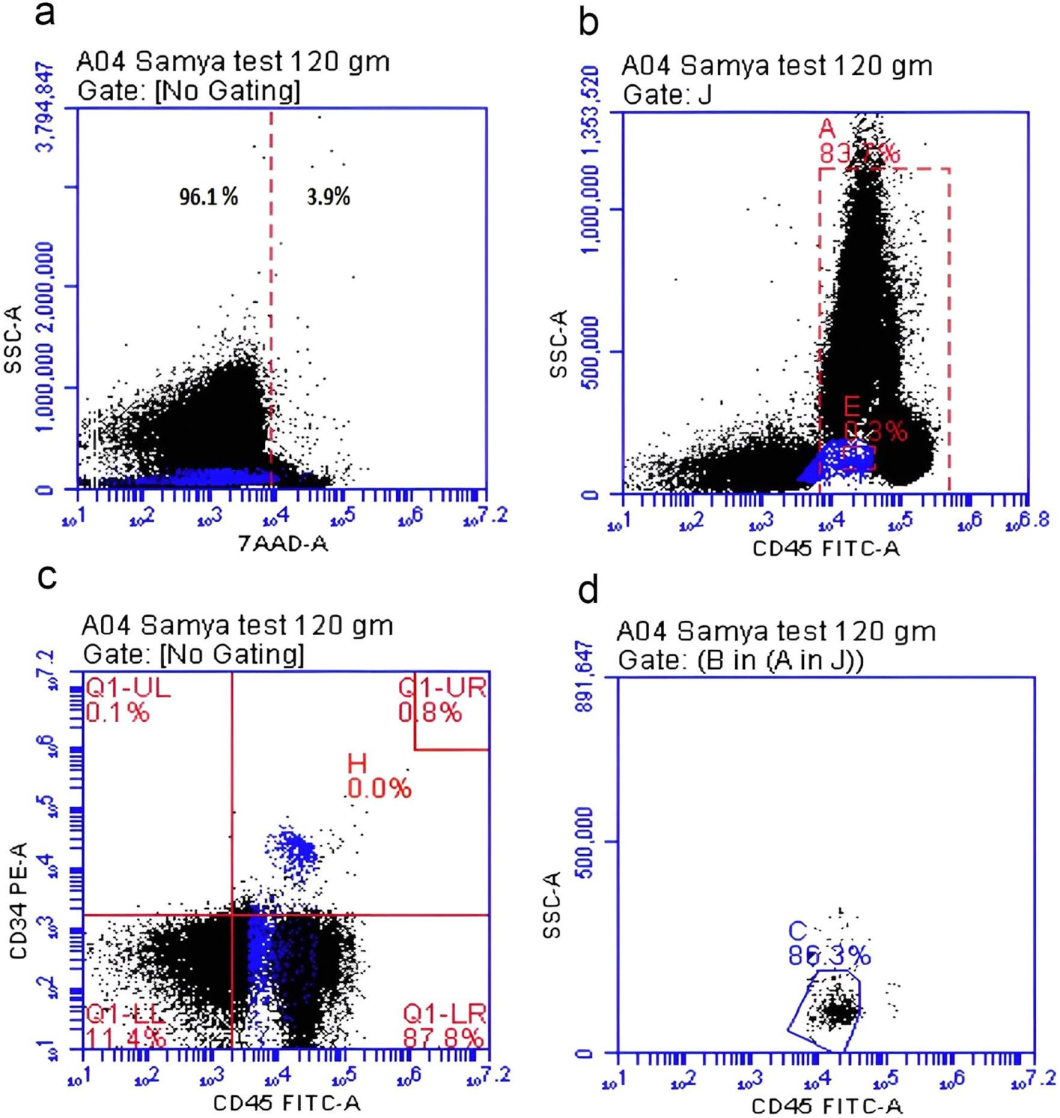
Gating and detection of CD34^+^ cells by flowcytometry. (**a**) Identification of cell viability using 7-Amino-Actinomycin D (7-AAD), a fluorescent intercalator dye that can selectively bind to DNA in dead cells, (**b**) Gating of live CD45 + hematopoietic cells (83% of viable cells), (**c**) Gating of double positive cells CD45 + /CD34 + cells (0.8% of cells), (**d**) Identification of the hematopoietic stem cells population CD34 + /CD45 + dim cells (86.3% of double positive for CD45^+^ and CD34^+^).

The data were analyzed using the software System (Becton, Dickinson, and Company). The Stem-Kit™ Reagents (Beckman Coulter, USA) were used. Histograms were used to characterize CD34 + /CD45 + dim hematopoietic stem cells by Flow Cytometry according to the International Society of Hematotherapy and Graft Engineering (ISHAGE) Guidelines^18^. The clinical pathologist and the technical person who measured the HPCs were blinded regarding the study groups. Gating was done in four steps as illustrated in Fig. 1. (a) Identification of cell viability using 7-Amino-Actinomycin D (7-AAD), a fluorescent intercalator dye that can selectively bind to DNA in dead cells. (b) Gating of live CD45 + hematopoietic cells; (c) Gating of double positive cells CD45 + /CD34 + cells; (d) Identification of the hematopoietic stem cells population CD34 + /CD45 + dim cells. The absolute count of CD34^+^ hematopoietic stem cells was calculated by multiplying the obtained percentage of CD34^+^ by the absolute *TNC*/μL as per ISHAGE Guidelines^18^.

### Power of the study

Power was calculated by the Stata Statistical Software (Stata Corp LLC, 2021, Release 17. College Station, TX). In the current study, the newborn total blood count in group 1 was 14672 ± 6098.2 cells/µL, while the total blood count in group 2 was 20366 ± 19479.8 cells/µL. Thus, using the Wilcoxon- Mann Whitney model, with power of 83.5% and α error 5%, we aimed to enroll 50 participants per group to detect the statistical difference between two groups.

### Randomization

When delivery was imminent (expected within 10 min), the nurse opened a sealed, numbered, opaque envelope containing the treatment allocation deigned by random allocation sequence generated by Microsoft Excel^19^. The included babies were randomly assigned into 2 groups: Group 1: babies were subjected to DCC 30 s after birth (50 newborns), Group 2: babies were subjected to DCC 60 s after birth (53 newborns).

### Statistical analysis

Statistical analysis was performed using commercially available software, Statistical package for Social Science (IBM Corp. Released 2017. IBM SPSS Statistics for Windows, Version 25.0. Armonk, NY: IBM Corp.). Mann Whitney Test was used to assess the statistical significance of the non-parametric variable difference between two study groups. Chi-Square test was used to examine the relationship between two qualitative variables. Fisher’s exact test was used to examine the relationship between two qualitative variables when the expected count is less than 5 in more than 20% of cells.

Correlation was done using Spearman’s correlation. Linear regression analysis was used for prediction of risk factors, using generalized linear models. Univariate regression was used to examine the effect of a single independent variable while in multivariate regression analysis, more than one variable is analysed together for association or interactions, to explore which of the independent variables are independently associated with the outcome. All reported *p* values were two-tailed and *p* < 0.05 was considered statistically significant.

### Ethics approval and consent to participate

This study was in line with the principles of the Declaration of Helsinki and approved by Mansoura Faculty of Medicine Institutional Research Board (MS/17.02.80). We obtained informed consents from all mothers prior to delivery.

### Consent to participate

Written informed consent was obtained from mothers prior to inclusion of their babies and cord blood sampling in the study.

## Results

**Figure 2:**
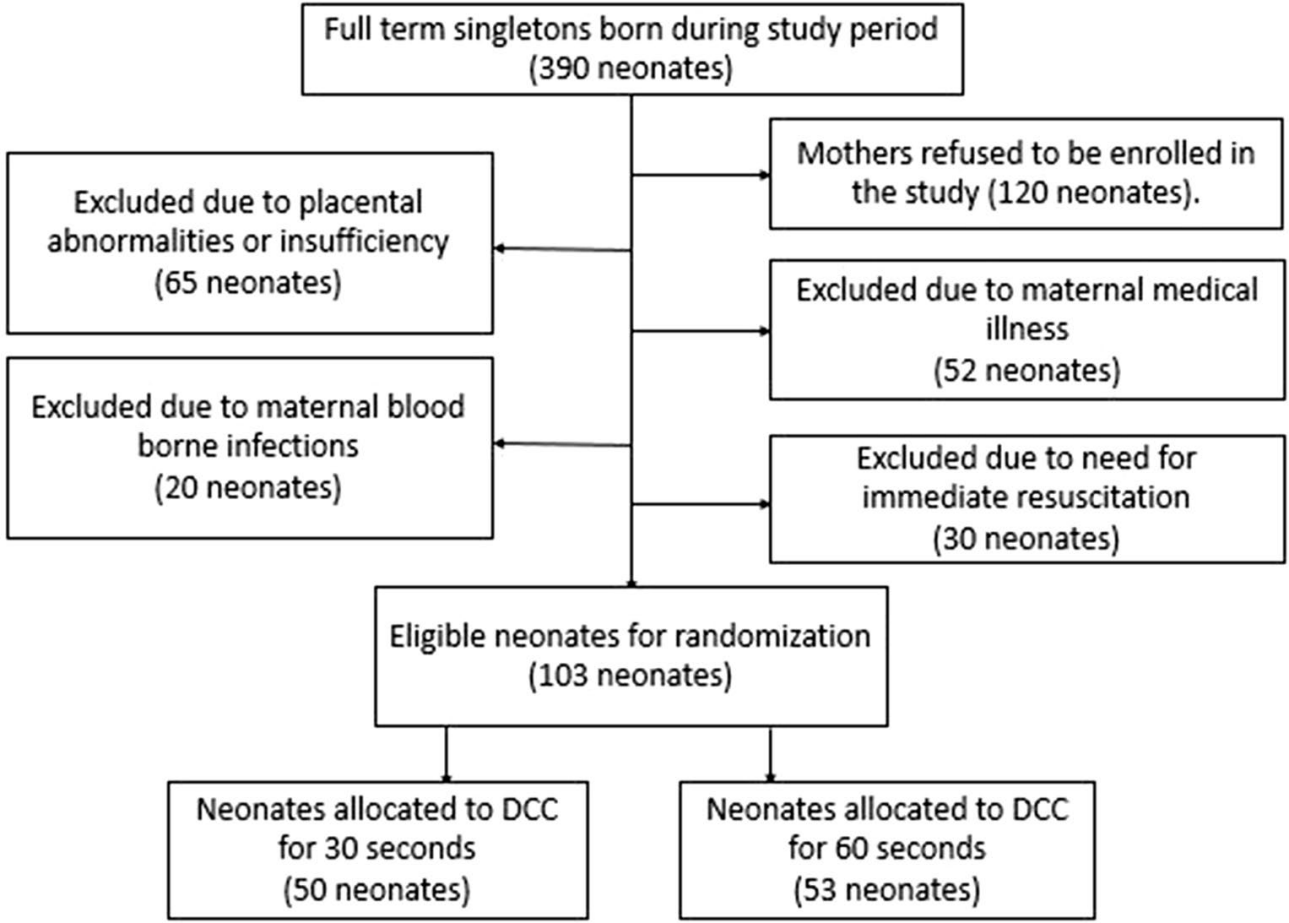
Diagram showing the flow of participants through stages of the study. DCC, delayed cord clamping. DCC, delayed cord clamping.

Among 390 full term neonates who were delivered by elective CS in our hospital during the study period, 120 neonates were excluded due to maternal refusal to be enrolled in the study. In addition, 65 neonates had sonographic evidence of placental insufficiency, 52 neonates had maternal illness, 20 neonates had maternal blood-borne infections and 30 ones required major resuscitative measures such as intubation. Finally, 103 neonates were included in the study. The Flow of participants through stages of the study is shown in Fig. 2.

Both groups in the current study were matched regarding their baseline neonatal and maternal characteristics as shown in Table 1. There was no statistically significant difference between neonates in both study groups regarding the birth weight, gender, fetal heart rate, post-natal feeding either breast or artificial milk, incidence of post-natal infections and platelet count. However, group 2 had higher hemoglobin, hematocrit, TNC and CD34^+^ cells than group 1 with *p* values 0.003, 0.03, 0.049 and 0.035, respectively. Regarding the maternal data, no significant difference was found regarding maternal age, gravidity, parity, indications of CS or maternal hematological parameters as hemoglobin, TNC or platelet count.

**Table 1:** Neonatal and maternal characteristics.

Parameters	Group (1) (No. = 50)	Group (2) (No. = 53)	*p* value
Neonatal characteristics			
GA (weeks) Median (IQR)	38 (37–40)	38 (37–40)	0.19
BW(Kg) Median (IQR)	3.3 (2.2–4.4)	3.5 (2.5–4.5)	0.20
Gender No. (%)			0.21
Male	23 (46)	18 (34)	
Female	27 (54)	35 (66)	
Fetal HR Median (IQR)	135 (120–145)	130 (120–145)	0.52
Feeding No. (%)			0.47
Breast milk	44 (88)	44 (83)	
Artificial formula	6 (12)	9 (17)	
Postnatal infection No. (%)			1.00
Yes	1 (2)	2 (3.8)	
No	49 (98)	51 (96.2)	
TNC of baby (cells/µL) Median (IQR)	13,000 (5600–36,000)	15,000 (7900–125,000)	0.049
CD34^+^ level (cells/µL) Median (IQR)	4580 (780–30,500)	6800 (1400–55,200)	0.035
HB (gm/dL)	14.7 (12.9–16.5)	15.7 (14–17.4)	0.003
Hct (%)	42.2 (36.6–47.8)	44.6 (38.8–50.4)	0.03
Platelets (cells /µL)	265,960 (207,455–324,465)	273,940 (207,822–340,058)	0.5
Maternal characteristics			
Mothers’ age (years) Median (IQR)	27.5 (19–42)	28 (18–41)	0.52
Gravidity and parity No (%)			0.91
Primigravida primipara	6 (12)	6 (11.3)	
Multigravida multipara	44 (88)	47 (88.7)	
Indications for CS No (%)			0.91
Previous CS	44 (88)	47 (88.7)	
Cephalopelvic disproportion	6 (12)	6 (11.3)	
Antenatal steroids use No (%)	27 (54)	32 (60.4)	0.513
Received antenatal steroids No (%)			0.892
2 Doses	8 (29.6)	10 (31.2)	
4 Doses	19 (70.4)	22 (68.8)	
Maternal HB (gm/dL) Median (IQR)	11 (8.5–12.5)	10.1 (8.6–12)	0.85
Maternal TNC (cells/µL) Median (IQR)	9000 (4000–18,000)	8000 (5000–14,000)	0.317
Maternal PLT (cells/µL). Median (IQR)	211,000 (126,000–390,000)	222,000 (111,000–402,000)	0.64

Mann Whitney Test was used to compare median and IQR between 2 groups, chi square test were used to compare percentage between 2 study groups, *p* value < 0.05: significant, No., number of the subjects, GA, gestational age, BW, birth, weight., HR, heart rate, IQR, interquartile range, No, number of the subjects, HB, hemoglobin, PLT, platelets, TNC, Total Nucleated Cells, CS, cesarean section.

**Figure 3:**
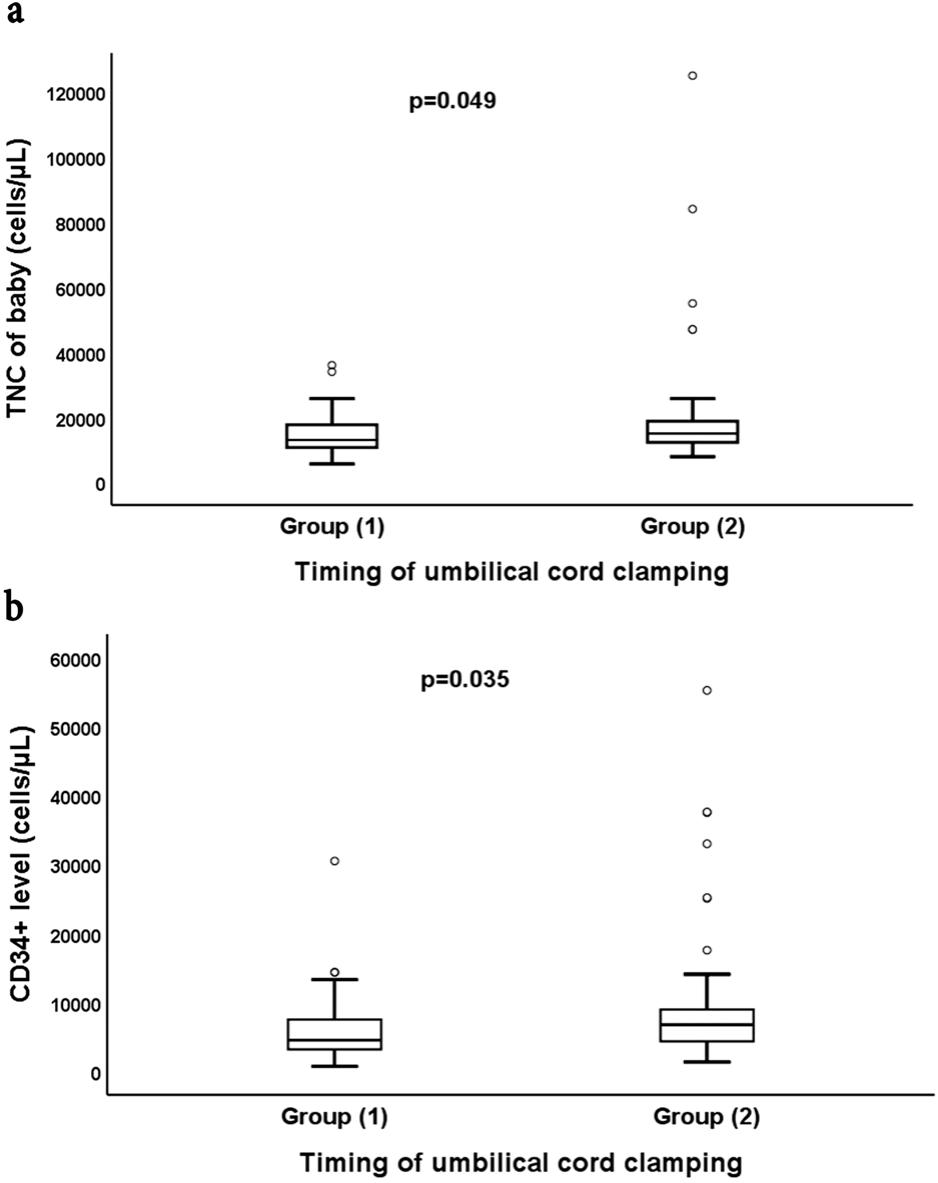
Boxplots comparing the TNC and CD 34^+^ cells count in both groups. The boxes are limited by the 75th and 25th percentiles of the data. The middle lines represent the median. (**a**) Box plot showing higher median value of TNC among group 2 than group 1, (**b**) showing higher median value representing CD 34 + cells in group 2 then group 1. Data are presented as median and interquartile range, *p* value determined by Z = Mann–Whitney test for group comparison, *p* value < 0.05: significant. Group 1: DCC for 30 s, group 2: DCC for 60 s, TNC: Total Nucleated Cells.

The primary outcome of the present study was to compare TNC and CD34^+^ cells as a maker of hematopoietic stem cells between both groups. Both were higher in group 2 compared to group 1, *p* value 0.049 and 0.03 respectively, presented in box plot in Fig. 3. Figure 3a represents the TNC count and 3b represents the CD34+ cells in both groups.

Regression analysis was conducted for prediction of factors affecting TNC and CD34 of baby, such as maternal age, gravidity, parity, gestational age, birth weight, maternal TNC and time of cord clamping, as illustrated in (Supplementary Table 1). However, it was found that GA and timing of cord clamping were the only significant confounders.

Older GA and cord clamping at 60 s were considered favorable predictors for increased TNC and CD34^+^ of baby in univariable and multivariable analyses as illustrated in Table2.

**Table 2:** Regression analysis for prediction of factors affecting TNC and CD34^+^ cells of baby.

		TNC				CD34			
		Univariable		Multivariable		Univariable		Multivariable	
		*Β*	*P*	*Β*	*P*	*β*	*P*	*β*	*p*
Cord clamping time	30	Reference		Reference		Reference		Reference	
	60	0.328	**0.001**	0.185	**0.038**	0.470	**0.001**	0.286	**0.046**
GA		0.355	** < 0.001**	0.330	** < 0.001**	0.406	**< 0.001**	0.352	**< 0.001**

β, Standardized Coefficients beta, TNC, Total Nucleated Cells, GA, gestational age. Significant values are in bold.

**Figure 4:**
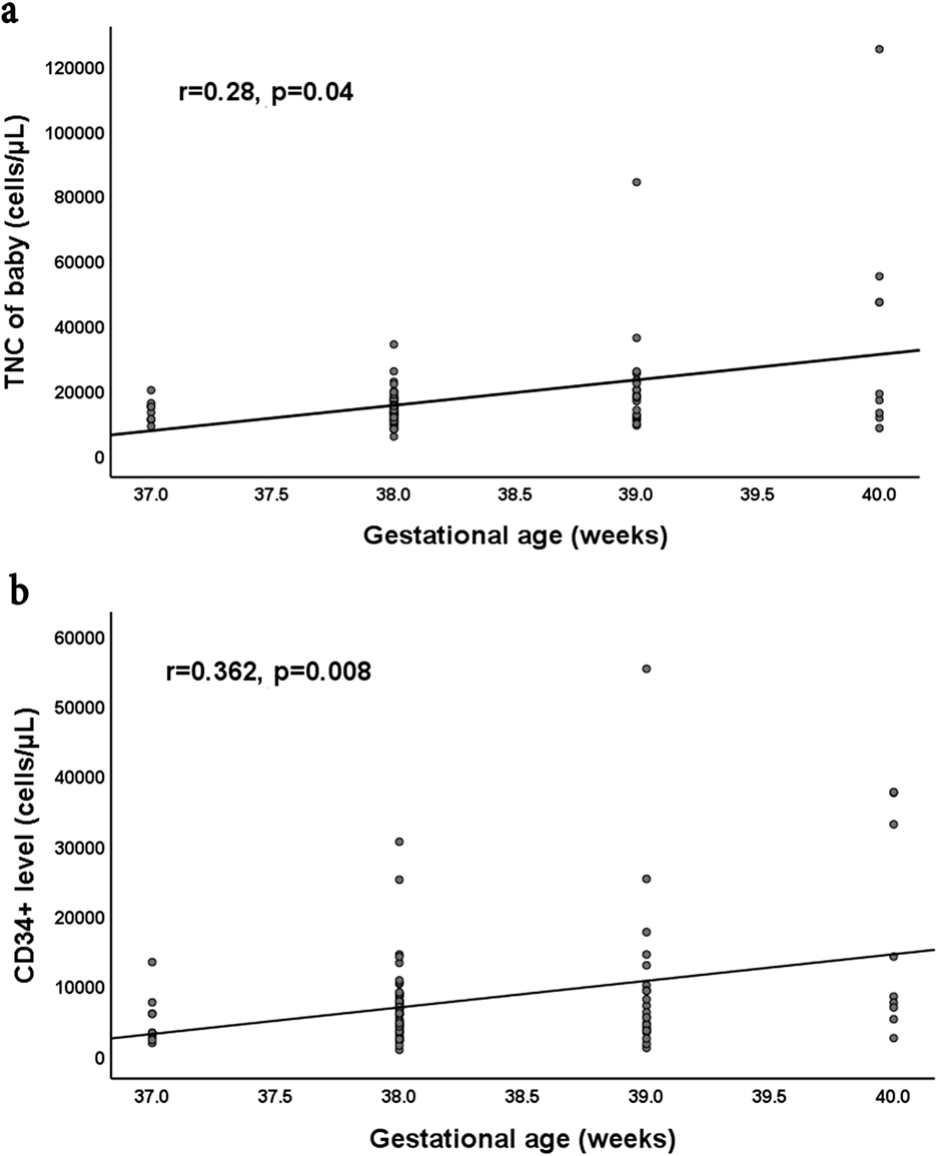
Correlation between gestational age (weeks) and both TNC and CD34^+^ cells (cells/µL). Scatter plot is presented to show the correlation. (**a**) Show increase in the TNC along with increasing the gestational age (GA), (**b**) show increase in the CD 34 + cells count with increasing GA. Results are presented as correlation coefficients (Spearman’s rho) and *p* < 0.05: significant, TNC, Total Nucleated Cells.

Regarding the correlation between gestational age and both the TNC and CD34^+^ cells, there was a significant positive correlation between the gestational age and both neonatal TNC and CD34^+^ cells, as illustrated in the scatter plot in Fig. 4. Figure 4a represents the correlation with TNC count and 4b represents the correlation with CD34+ cells.

## Discussion

Although DCC is the standard recommended practice in delivery room management, still the definition or the optimal timing of delay needed to achieve maximal placental transfusion has yet to be established through more and more studies^19^. The present study was conducted to describe the effect of different timings of DCC on CD34^+^ stem cells transfer to the baby. To the best of our knowledge, no previous studies compared the impact of different timings of DCC on CD34^+^ cells yield in the full-term babies.

The neonates in group 2 had higher TNC and hematopoietic stem cells (CD34^+^ cells) compared to those in group 1. This may be attributed to prolonged placental transfusion in the newborns subjected to more delay in umbilical cord clamping as in group 2. It is presumed that DCC allows physiologic transfer of residual placental blood to the infants^20^. In FT infants, DCC for 60 s after birth can add about 80 mL of placental blood to the infant’s circulation that can increase up to 100 mL if the delay extended to 180 s^21^. The practice of DCC is not an interventional treatment, but it can be considered the standard practice respecting natural and physiological redistribution of blood between the placenta and the infant in 1st few minutes after birth. This actually allows smooth transition from the placental to pulmonary circulation as well as autotransfusion of fetal blood highly enriched with stem cells and immunoglobulins that provides maximal neonatal benefits^22,23^.

Our results are like another recent study from our center that concluded that DCC led to higher CD34^+^ stem cells transfusion in PT babies with placental insufficiency. Also, they had higher levels of HB and Hct without significant additional risk of polycythemia or jaundice among those infants^24^.

Regarding other hematological parameters, we found that the babies in group 2 had significantly higher hemoglobin and hematocrit compared to those in group 1. This was in agreement with that reported from many studies that DCC (60 s after birth) achieved higher hemoglobin and hematocrit levels soon after birth and up to 6 months after birth^25^. Similar results were found in preterm babies with DCC (30–180 s after birth)^26^.

The Australian placental transfusion study (APTS) is the largest clinical trial comparing ECC versus DCC in PT infants less than 32 weeks, with cord clamping at 10 s and 60 s, respectively after birth. It concluded that DCC did not decrease the mortality or major morbidity at 36 weeks corrected gestational age^27^. However, long-term follow up of those infants for 2 years revealed that relative mortality was reduced by 30% in DCC group, with no difference regarding major disability^28^.

In the present study, univariable and multivariable regression analyses revealed that older GA and delayed cord clamping at 60 s were considered favorable predictors for higher TNC and CD34^+^ in the full-term neonates. Advanced GA may be associated with higher birth weight that was found to be associated with higher TNC in another study^29^. Our cohort had higher birth weight in delayed cord clamping for 60 s, however it did not reach statistically significant value.

Although a significant positive correlation was found between the gestational age and both the TNC count and CD34^+^ cells in our cohort, a negative correlation was found between gestational age and CD34^+^ cells in premature infants (between 25 and 37 weeks), in another study that concluded that PT babies had higher CD34^+^ cells despite of having lower TNC count than FT babies^30^. The higher levels of CD34+ HSPCs in PT cord blood may be attributed to the fluctuating cytokine and chemokine levels that stimulate the transfer of hematopoiesis from the liver to the bone marrow in PT newborns^31^.

There was significantly higher CD34^+^ cells yield and TNC count with older gestational age. This positive correlation can be related to increased birth weight and better placental transfusion with older GA^29^. In addition, it can support the current recommendation regarding elective cesarean delivery (ECD) to be at 39 weeks^32,33^. It can be concluded that the best yield of CD34^+^ stem cells can be obtained from DCC after 60 s together with delayed elective CS till 39 weeks unless in emergency settings.

In conclusion, DCC for 60 s achieved the best placental transfusion of CD34^+^ stem cells as well as higher hemoglobin and hematocrit values. Hence, it is the standard procedure for all newborns who do not require immediate resuscitation.

The limitations of the current work lie in the fact that it is a single center study on a relatively small number of newborns, and it lacks a control group with immediate cord clamping. However, we found it unethical to perform immediate clamping while DCC is the standard practice. Further multicenter studies on a larger number of both preterm and full-term neonates will be valuable. Additionally, long term follow-up of the effect of DCC on neurodevelopmental outcome is a valuable area of future research.

### Supplementary Information

Supplementary Table 1.

## Data Availability

The datasets used and/or analysed during the current study are available from the corresponding author on reasonable request.

## Abbreviations

AAP: American Academy of Pediatrics
ACOG: American College of Obstetricians and Gynecologists
AHA: American Heart Association
BW: Birth weight
CBU: Cord blood unit
CBC: Complete blood count
dL: Deciliter
DCC: Delayed cord clamping
ECC: Early cord clamping
ECS: Elective cesarean section
FT: Full term
GA: Gestational age
HIV: Human immunodeficiency virus
HB: Hemoglobin
Hct: Hematocrit
HSCs: Hematopoietic stem cells
MUH: Mansoura University Hospital
NRP: Neonatal Resuscitation Program
Plt: Platelet
PT: Preterm
RCT: Randomized clinical trial
SPSS: Statistical package of the social science
TNCs: Total nucleated cells
UC: Umbilical cord
UCB: Umbilical cord blood
