# Supplementary material for: Effect of different timings of umbilical cord clamping on the level of CD34+ cells in full-term neonates
Source: Sci Rep. 2023 Dec 21;13:22917. doi: 10.1038/s41598-023-50100-9 (PMC10739938; doi:10.1038/s41598-023-50100-9)
Supplement: Supplementary file 1 — Supplementary Table 1. [file 41598_2023_50100_MOESM1_ESM.docx]

**Table 1: Correlation of gestational age, birth weight, maternal TNC and neonatal order of birth to TNC and CD34+ cells in both groups. (supplementary)**

| Parameters | Group (1)  (No. =50) | Group (2)  (No. =53) |
| --- | --- | --- |
|  | **TNC** | |
| Gestational age (Ws) | **r=** 0.255  p=0.07 | **r=** 0.281  p=0.04* |
| Birth weight (Kg) | **r=** -0.147  p=0.30 | **r=** -0.314  p=0.02* |
| Maternal TNC  (cells/µL) | **r=** 0.025  p=0.86 | **r=** 0.274  p=0.04* |
| Maternal age | r= -0.012  p= 0.9 | r= 0.02  p=0.8 |
| Gravidity | r= -0.4  p= 0.009** | r= 0.06  p= 0.7 |
| Parity | r= -0.232  p= 0.1 | r= -0.09  p= 0.51 |
|  | **CD34^+^ cells** | |
| Gestational age (37-40ws). | **r=** 0.120  p=0.405 | **r=** 0.362  p=0.008^**^ |
| Birth weight (2.5-4kg) | **r=** -0.128  p=0.37 | **r=** -0.284  p=0.03^*^ |
| Maternal age | r= 0.03  p=0.8 | r= -0.04  p= 0.8 |
| Gravidity | r= -0.27  p= 0.05* | r= -0.2  p= 0.06 |
| Parity (Primigravida versus multipara) | r= -0.168  p=0.242 | r= -0.330  p=0.01^**^ |

Results are presented as correlation coefficients (Spearman’s rho) and * P ≤ 0.05: significant, TNC: Total Nucleated Cells.
